# Improvement of the ability to recover balance through versatile kinesthetic learning experiences

**DOI:** 10.3389/fspor.2022.975304

**Published:** 2023-01-17

**Authors:** Yuki Matsuura, Masahiro Kokubu, Yosuke Sakairi

**Affiliations:** ^1^ Faculty of Education, Utsunomiya University, Utsunomiya, Japan; ^2^ Faculty of Health and Sport Sciences, University of Tsukuba, Tsukuba, Japan

**Keywords:** skill acquisition, learning method, balance recovery, motion analysis, implicit learning

## Abstract

The purpose of the present study was to compare learners' movement variability while maintaining balance and the ability to recover balance using the kinesthetic-experiential learning (KEL) method of implicit learning and the model-mastery learning (MML) method of explicit learning. The participants were 29 healthy university students. They were randomly divided into two groups (KEL and MML). They were required to balance both knees on an exercise ball. The balancing time and the ability to recover their balance were measured using motion capture. Results indicated that balancing time was significantly improved for both learning methods. Regarding the learners' movements while maintaining balance, they maintained balance while moving in the KEL method, whereas they maintained balance by keeping the entire body stationary in the MML method. Concerning the ability to recover, the KEL method improved the balance recovery ability more effectively than the MML method. Therefore, we concluded that using the KEL method at the initial stage of learning improves learners' balance recovery ability and increases movement variability.

## 1. Introduction

Learners use various sensory cues in learning motor skills. Although implicit and explicit learning have been compared to illustrate these sensory cues, the debate concerning which is more effective in kinesthetic learning has yet to be settled. Previous studies have reported that the effectiveness of implicit or explicit learning also depends on the proficiency levels of learners (1, 2) and the nature of the motor task (3, 4).

Balance tasks are a type of motor task for which implicit learning has proven effective. Orrell et al. (5) reported that implicit learning was effective in a balance task requiring participants to maintain an upright posture on top of a wooden platform mounted freely on a horizontal beam. This study used two alternative strategies to encourage implicit learning: learning by analogy (6, 7) and learning without errors (8). Various types of sensory feedback, such as the synthesis of vestibular input and visual and somatic sensations, are used in learning to enhance balance ability (9). Therefore, some learners may take time to become aware of crucial sensory cue information, making it necessary to devise ways to promote implicit learning. However, it may be challenging to eliminate errors in tasks requiring dynamic and advanced balancing abilities, and safety must also be considered. Measures must be taken to safely and effectively ensure that learners are aware of important sensory cues to utilize implicit learning in such tasks.

**Figure 1:**
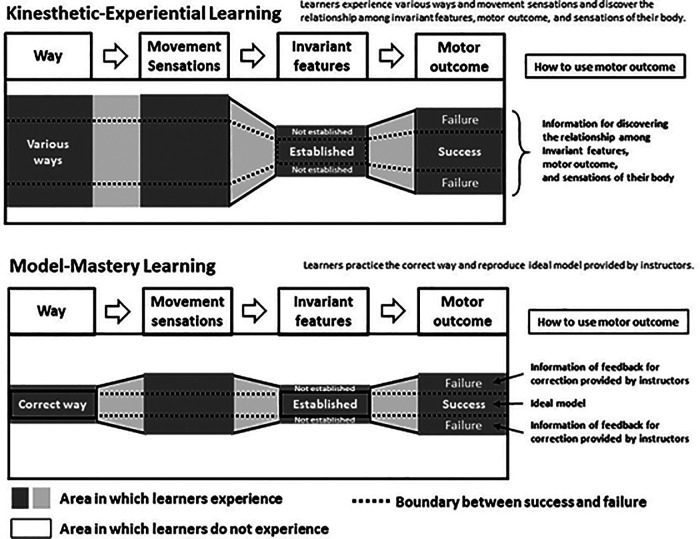
Conceptual diagram of kinesthetic-experiential learning and model-mastery learning methods (Matsuura et al., 2022). Specific details are shown in Tables 1, 2.

Invariant features are one type of crucial sensory cue information used when learning movements that make dynamic use of the entire body. Invariant features emerge from interaction with objects in the environment when a specific motion is repeated, which influences task achievement (10). Humans use invariant features generated from body–environment interactions to perform and control their actions (11). A strategy for learning by utilizing invariant structures is called “the global dynamics approach” in a robot's motor learning (12). Matsuura et al. (13, 14) applied a global dynamics approach to humans and proposed the kinesthetic-experiential learning (KEL) method as an implicit motor learning technique that makes use of invariant features (Figure 1 and Table 1). It has been reported that learning using the KEL method makes it possible for learners to carry out implicit learning safely and effectively (14).

**Table 1:** Two types of learning methods for skill acquisition; Kinesthetic-Experiential Learning and Model-Mastery Learning methods (14).

Kinesthetic-Experiential Learning	Model-Mastery Learning
**Elements to emphasize in order to establish invariant features**	
Emphasizing the sensations of learners' body when establishing invariant features	Emphasizing the way to establishing invariant features
Instructors emphasize which body parts the learners should pay attention to and let them feel the sensation of their body	Instructors emphasize the correct way of moving their body by demonstrating ideal model
**Instructions to have learners establish invariant features**	
Instructors allow learners to experience various ways of moving their body to implicitly learn the relationship between invariant features and motor outcome by accumulating their own experience.	Instructors explicitly provide learners with the way to move their body so that the invariant features are established and have the learners repeatedly practice.
Instructors do not explicitly indicate an ideal model or the correct way of moving their body	Instructors explicitly indicate an ideal model as well as the correct way of moving their body
**Approaches that learners use to establish invariant features**	
Learners discover the relationship among invariant features, motor outcome, and sensations of their body from their experiences	Learners are provided with the relationship among invariant features, motor outcome, and movements of their body by the instructor and reproduce it
Learners can try various and flexible ways	Learners only try a fixed way

**Table 2:** Specific instructional content of Kinesthetic-Experiential Learning and Model-Mastery Learning methods in the present study.

	Kinesthetic-Experiential Learning	Model-Mastery Learning
**Invariant features** **in the present task**	“The relationship between the center of gravity of a learner and the center of the ball. As a condition to establish the task, “COM is on BOS” is a basic requirement to maintain balance (17).”
**Elements to emphasize in order to establish invariant features**		
**Instructions regarding invariant features**	“Feel” your center of gravity (i.e. midpoint of the waist) and the center of the ball	“Align” the center of the ball and your center of gravity (i.e. midpoint of the waist)
**Instructions to have learners establish invariant features**		
**Procedure to ride on the ball**	Ride on the ball on all fours. Pay attention to the sensation related to the exercise ball and align your center of gravity and the center of the ball. Then, repeat falling on the soft mat.	Ride on the ball with the appropriate foot width (about two fists). The assistant takes the learner’s hand and guides him/her to align the center of the ball with the point just above the learner's pelvis (the same procedure was used when the measurement was conducted).
**Postural variability**	From crawling on all fours, try different postures on the ball. (For example, raise one hand or one leg from the crawl position.)	Once the balance becomes stable after the procedure on the ball, the learner releases the hand from the assistant and maintains the balance by himself/herself. The learner keeps arms extended to sides and faces forward to maintain a stable balanced posture. Imagine that the knees, hips, and shoulders are aligned with (in a straight line with) the center of the ball.
**What to do when out of balance**	The learner falls on the soft mat (because s/he has already experienced falling safely when riding on the ball). As one of the experiences of various sensations, the learner intentionally loses balance in various directions and experiences falling on a soft mat. This is done at the stage of postural variability.	The assistant helps the learner by giving support, so that the learner does not fall.

BOS, Base of Support; COM, Center of Mass.

Matsuura et al. (13, 14) compared the learning effects of implicit learning (KEL method) to those of explicit learning [model-mastery learning (MML) method] (Figure 1 and Table 1) using a balancing task on an exercise ball. Results revealed that the MML method was more effective for postural stability and enhanced performance in balancing time, while the KEL method was more effective for recovering balance. However, this was a subjective evaluation by observers, and motion analysis on the learners was not performed. Therefore, the strategy employed by learners to maintain and recover their balance remains unclear, as does the extent to which their ability to recover their balance improved. The basic postural control ability to adjust the imbalance is an important basic ability in any exercise (15). These facts suggest that it is important to conduct motion analysis to measure the effects of each learning method on the ability to balance.

Hence, this study conducted motion analysis to determine the difference between the movement while maintaining balance and the ability to recover balance when using the KEL method vs. the MML method. We developed the following hypotheses: (1) The ability to maintain balance increases with either learning method; (2) Movement variability while maintaining balance differs between the KEL and MML methods; and (3) The KEL method leads to a higher ability to recover balance than the MML method.

## 2. Method

### 2.1. Participants

The participants were 29 healthy university students from University A (5 men, 24 women; average age: 19.66 years, *SD* = 0.66) who belonged to departments that did not specialize in physical education. In addition, they had no previous experience with the exercise tasks used in this study. The participants were randomly divided into two groups: the KEL group consisted of 15 participants (3 men, 12 women, average age: 19.60 years, *SD* = 0.80), and the MML group consisted of 14 participants (2 men, 12 women, average age: 19.71 years, *SD* = 0.45).

### 2.2. Apparatus

**Figure 2:**
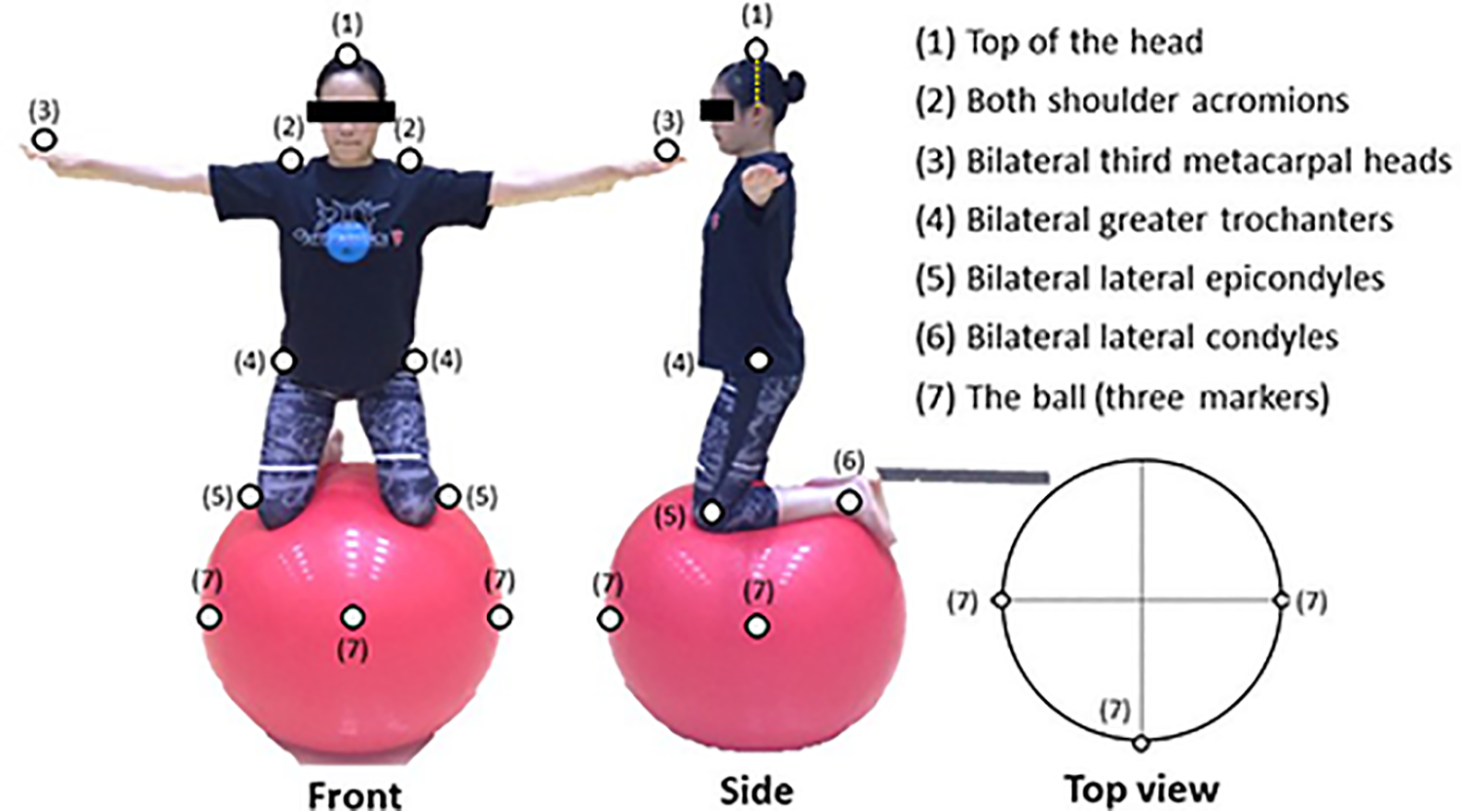
Locations of infrared reflection markers. Infrared reflective markers for motion capture were attached at 11 points to each participant. The three markers were attached to the exercise ball. The numbers in parentheses beside the markers correspond to the numbers indicating body parts on the right side of the figure.

Ten optical motion capture cameras (Optitrack Flex13: Naturalpoint, 120 Hz) were used to measure balancing time and the ability to recover balance, and the three-dimensional coordinates of each marker were calculated. Infrared reflective markers for motion capture were attached to 11 points on each participant (see Figure 2): top of the head, both shoulder acromions, bilateral third metacarpal heads, bilateral greater trochanters, bilateral lateral epicondyles, and bilateral lateral condyles. The three markers were attached to the exercise ball (85 cm in diameter; Gymnic, Inc.). In addition, four joint mats (Senoh Corporation), one soft mat (Senoh Corporation), a stopwatch, and two video cameras were used in the practice of the tasks.

### 2.3. Balancing task

**Figure 3:**
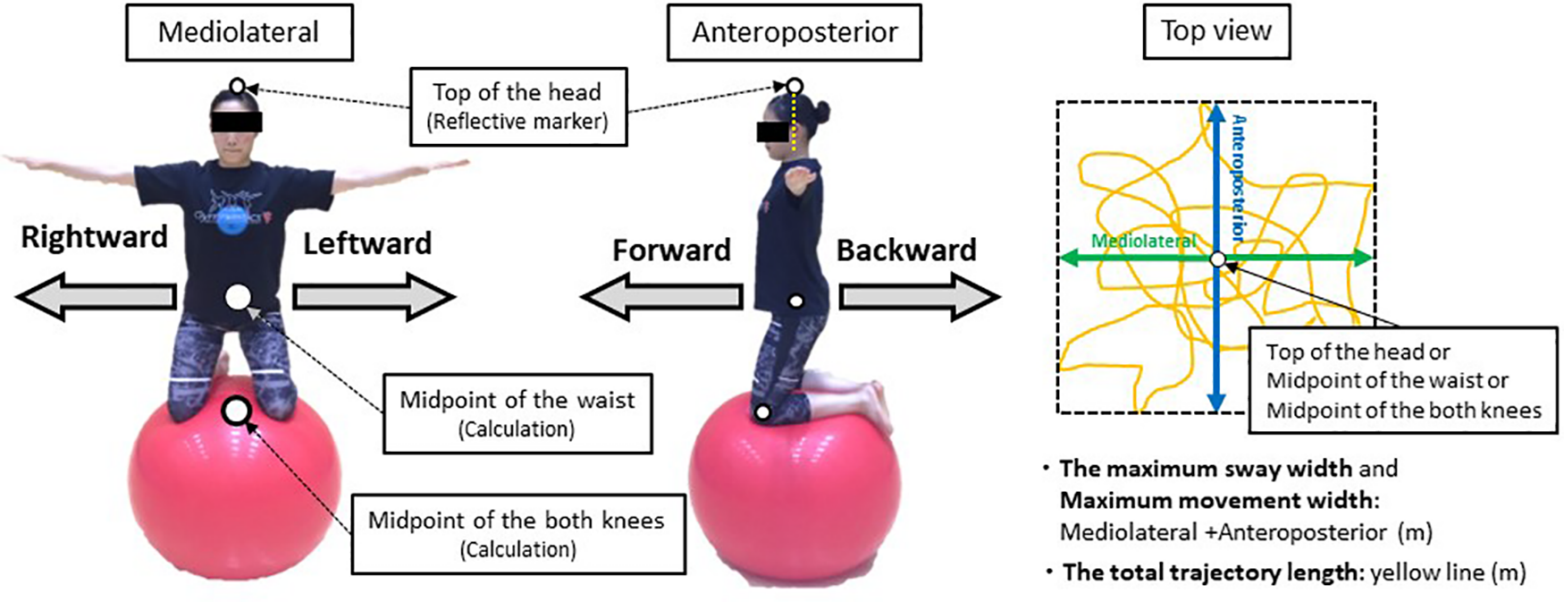
Parts and directions used to evaluate sway width in the balance maintenance test and movement width in the balance recovery ability test. The three parts (top of the head, midpoint of the waist, and midpoint of both knees) are used to evaluate sway width in the balance maintenance test. For the midpoint of the waist and midpoint of both knees, we calculated the midpoint from the left and right markers and used this value. In the balance recovery ability test, we compared the total maximum movement width in the anteroposterior and mediolateral directions of the midpoint of the waist.

**Figure 4:**
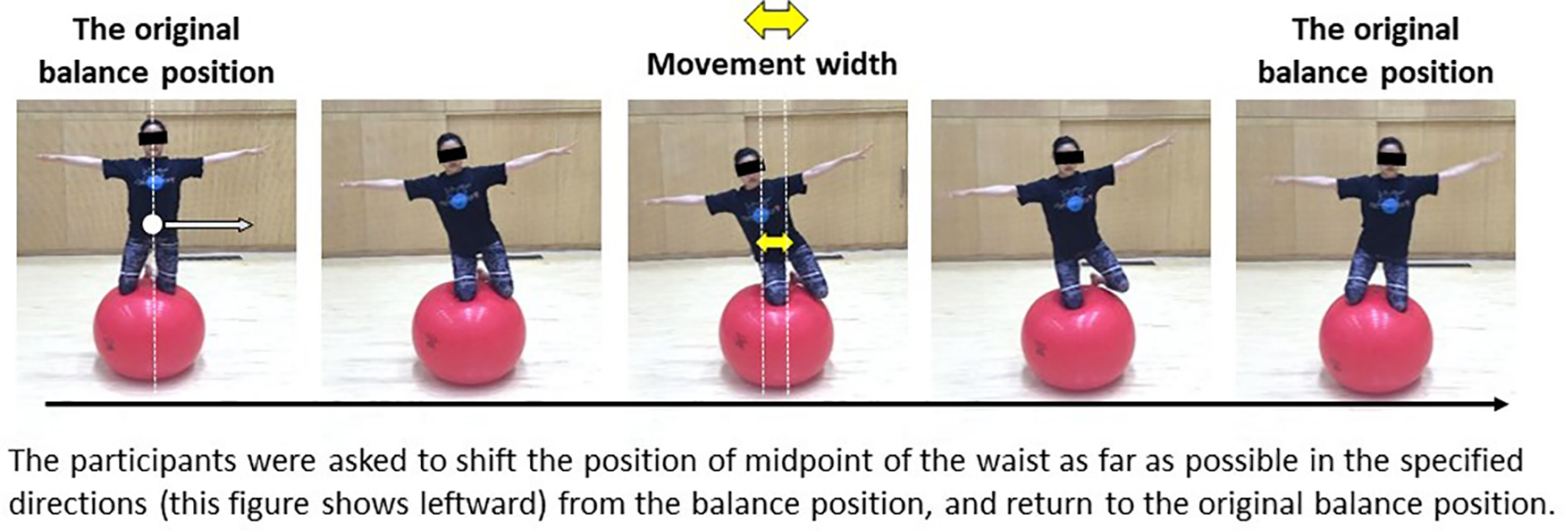
Test of balance recovery ability: left direction shifts task example. The width indicated by the yellow arrow is the movement width. Because the maximum movement width was defined as the sum of all maximum values in the anteroposterior and mediolateral directions, all maximum values were converted to an absolute value. The five trials were performed in each direction, totaling 20 trials.

As in Matsuura et al. (13), the balancing task (Figure 2) was also used in this study. It required participants to balance with both knees on an exercise ball. The goal of the task, in both groups, was to maintain this balanced posture for as long as possible. In addition, to measures their ability to recover balance, we evaluated how much participants could adjust their posture in the anteroposterior and mediolateral directions from the original balance position when balancing on an exercise ball and assessed the moving distance of the midpoint of the waist (Figures 3, 4). This task is similar to the Functional Reach Test (16), which measures the dynamic balance function as indices of limits of stability. The five trials were performed in each direction, totaling 20 trials. The analysis was performed from two viewpoints: maximum movement width and success rates.

### 2.4. Procedure

**Figure 5:**
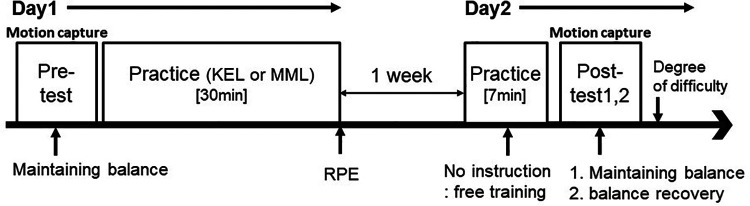
Experimental protocols. First, the participants performed a pre-test on maintaining balance using motion capture before practice was conducted. The participants were randomly divided into two groups (the KEL method and the MML method), and 30 min of balancing practice were conducted according to each learning method. The participants then responded to the RPE after the practice session. After 1 week, a second practice session was conducted for 7 min. After that, a post-test of maintaining balance was performed using motion capture. After the measurement, the test of balance recovery ability was presented, and a measurement was performed by motion capture without any practice time.

The participants were randomly divided into two groups using the KEL method and the MML method. Infrared reflective markers were attached to 11 points on each participant (see Figure 2), and a pre-test of maintaining balance before practice was conducted using motion capture. About 30 min of practice were then conducted according to each learning method to allow participants to practice balancing. After the practice session, the participants completed the ratings of perceived exertion (RPE). After 1 week, a second practice session was conducted for 7 min. After that, infrared reflective markers were then attached to each participant, and a post-test of maintaining balance was performed using motion capture. After the measurement, the test of balance recovery ability was presented, and a measurement was performed by motion capture without any practice time. The pre-test for the balance recovery ability was not performed to ensure safety as there was a high risk that participants would lose their balance and fall off the ball. The participants responded to the degree of difficulty after all measurements were completed. The research protocol is shown in Figure 5.

### 2.5. Learning methods

Table 2 shows a comparison of the specific content used in this study. The contents were decided based on discussions between one university faculty member who specializes in gymnastics coaching, one university faculty member who specializes in sports psychology, and one researcher who specializes in gymnastics. To ensure all participants received consistent teaching content, we only provided specific instructions, as shown in Table 2.

### 2.6. Measurements

#### 2.6.1. Japanese scale for RPE

To measure subjective exercise intensity, we used the Japanese version of a scale formulated by Onodera and Miyashita (18) based on the scale devised by Borg (19, 20). The scores on this scale range from 6 to 20. On the scale, descriptions were given for the odd numbers (i.e., “7: very easy” to “19: very hard”), and participants responded by choosing a number representing the exertion experienced while performing the task.

#### 2.6.2. Degree of difficulty of the task

To confirm that participants felt no difference in the degree of difficulty of the tasks between the learning methods, the participants were asked to rate the task of balancing on their knees and the applied task on a 5-point scale, ranging from 1 (very easy) to 5 (very difficult).

#### 2.6.3. Improvement of balancing skills

The following tests were conducted to measure the balancing skills.

##### 2.6.3.1. Test of maintaining balance: Balancing time

To examine the differences in the ability to maintain balance because of the variation in learning methods, balancing times were measured. In the starting position of the balance test, the assistant stood in front of the learner and supported them in the balanced position by holding both of their hands. The balancing time measurement began when the assistant released the learner's hands. Loss of balance was defined as: (1) the learner's foot reaching the floor (i.e., the moment when the ankle marker fell below 50 cm from the floor); (2) the learner's hand touching the ball (i.e., the moment the hand touched the ball); or (3) the learner holding on to the assistant (i.e., the moment the assistant and learner touched each other). The maximum balancing time was set to 1 min.

##### 2.6.3.2. Test of maintaining balance: Sway width and total trajectory length while maintaining balance

To examine the differences in movement while maintaining balance because of the variation in learning methods, the maximum sway width in the anteroposterior and mediolateral directions and the total trajectory length while maintaining balance were measured (Figure 3). Motion capture was used to calculate and compare the total sway width of the maximum movements of the top of the head, midpoint of the waist, and midpoint of both knees (i.e., midpoint of bilateral lateral epicondyles; see Figure 2). In addition, the maximum sway width in the anteroposterior and mediolateral directions was defined as the sum of all maximum values in the anteroposterior and mediolateral directions. Therefore, all maximum values were converted to an absolute value.

##### 2.6.3.3. Test of balance recovery ability: Maximum movement width of waist midpoint

To examine the differences in the learners' ability to recover balance because of the variation in learning methods, we measured the maximum movement width in the anteroposterior and mediolateral directions of the midpoint of the waist (see Figures 3, 4). Motion capture was used to calculate and compare the total maximum movement width in the anteroposterior and mediolateral directions of the midpoint of the waist. The maximum movement width was defined as the sum of all maximum values in the anteroposterior and mediolateral directions. Therefore, all maximum values were converted to an absolute value. Failure to return to the original balanced position after shifting the position of the midpoint of the waist was regarded as a failed attempt. In the test of balance recovery ability, if the movement was not successful at least once in five trials, the movement width was set to “0 m,” and the maximum movement width was calculated.

##### 2.6.3.4. Test of balance recovery ability: Success rates

To examine the differences in the success rates of the balance recovery ability tests because of the difference in learning methods, we calculated and compared the overall success rate of all four directions (right, left, front, and back). The five trials were performed in each direction, and a total of 20 trials were completed. Only trials with complete recovery to the original balance position were considered successful.

### 2.7. Analysis

For all data, a Kolmogorov-Smirnov test did not indicate that the data were consistent with a normal distribution, but a Levene's test indicated that the equality of the error variances was assumed at a significant level of 0.05. Therefore, in this study, we adopted a nonparametric test. A Mann-Whitney U test was used to derive RPE, degree of task difficulty, sway width, total trajectory length, balancing time, and maximum movement width of the midpoint of the waist to examine the difference between learning methods. Regarding the balancing time, pre- and post-balancing times were compared. Pearson's *χ*^2^ test was used to determine success rates of the test of balance recovery ability. Further, Cliff's *d* (21) was calculated for the RPE, degree of task difficulty, sway width, total trajectory length, balancing time, and maximum movement width. The phi-coefficient (*Φ*) was calculated for the success rates. All statistical analyses were performed using SPSS version 28.

## 3. Results

### 3.1. Japanese scale for RPE

A Mann-Whitney U test revealed that RPE scores were higher for the MML method (13.50, IQR: 12.75–14.25) than for the KEL method (12.00, IQR: 11.00–13.00) (*p* = .014, Cliff's *d* = 0.67).

### 3.2. Degree of task difficulty

A Mann-Whitney U test revealed no significant difference between the KEL and MML methods in degree of difficulty of the test of maintaining balance (KEL: 3.00, IQR: 2.00–3.00, MML: 2.00, IQR: 2.00–3.00) (*p* = .217, Cliff's *d* = 0.35) and the test of balance recovery ability (KEL: 4.00, IQR: 2.00–5.00, MML: 4.00, IQR: 3.00–4.25) (*p* = .813, Cliff's *d* = 0.11).

### 3.3. Improvement of balancing skills

#### 3.3.1. Test of maintaining balance: Balancing time

**Figure 6:**
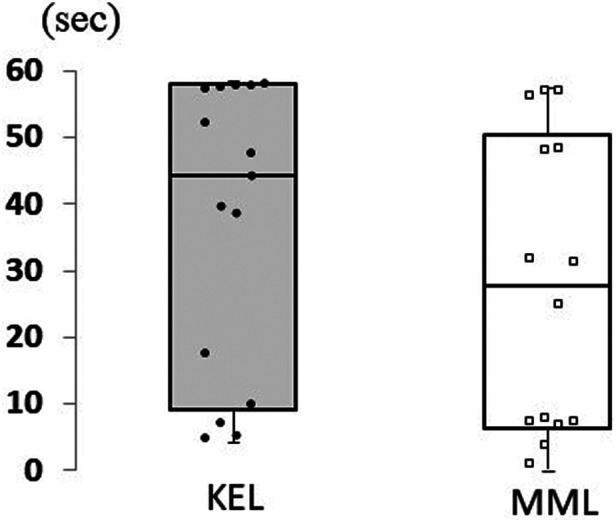
Difference in balancing time between KEL and MML methods. To examine the differences in the balancing time because of the differences in learning methods, the difference subtracted pre-balancing time from post-balancing time. No significant difference was found between the KEL and MML methods in terms of improved balancing time; it improved for both learning methods.

A Mann-Whitney U test revealed no significant difference between the KEL and MML methods in improved balancing time (*p* = .186, Cliff's *d* = 0.52) (Figure 6). These results support hypothesis 1.

#### 3.3.2. Test of maintaining balance: Sway width and total trajectory length while maintaining balance

**Figure 7:**
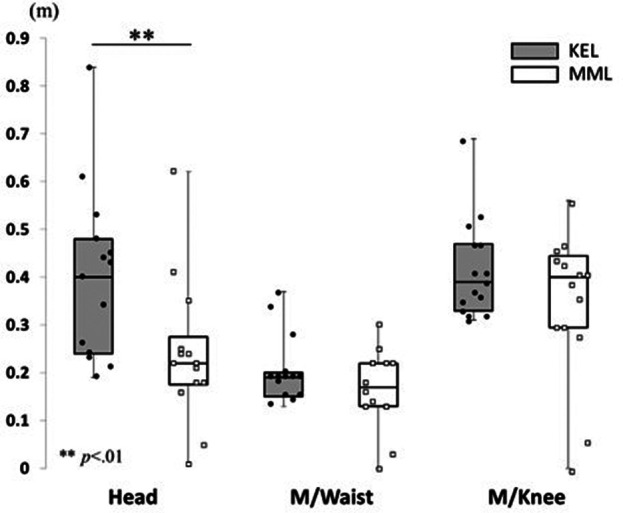
Difference in sway width while maintaining balance between KEL and MML methods. To examine the differences in motion while maintaining balance because of the differences in learning methods, the maximum sway width while maintaining balance was measured. A significantly larger sway width could be observed at the top of the head in the KEL method.

**Figure 8:**
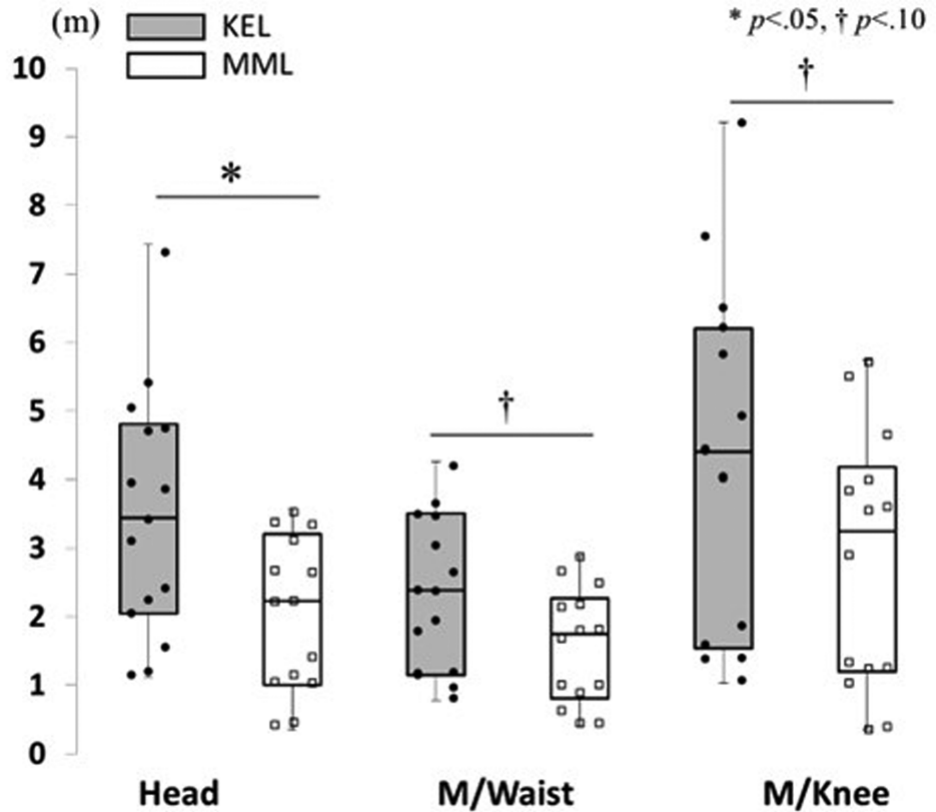
Difference in total trajectory length while maintaining balance between KEL and MML methods. To examine the differences in motion while maintaining balance because of the differences in learning methods, total trajectory length while maintaining balance was measured. A significantly larger total trajectory length could be observed at the top of the head in the KEL method.

A Mann-Whitney U test revealed a significantly larger sway width at the top of the head in the KEL method than in the MML method (*p* = .009, Cliff's *d* = 0.39) (Figure 7). No significant differences were observed in the sway widths of the midpoint of the waist (*p* = .477, Cliff's *d* = 0.02) or the midpoint of both knees (*p* = .477, Cliff's *d* = 0.05). In addition, the total trajectory length of the head was significantly larger in the KEL method than in the MML method (*p* = .018, Cliff's *d* = 0.59). Marginally significant differences were observed in the total trajectory length of the midpoint of the waist (*p* = .085, Cliff's *d* = 0.44) and the midpoint of both knees (*p* = .051, Cliff's *d* = 0.47) in the KEL method than in the MML method (Figure 8). These results support hypothesis 2.

#### 3.3.3. Test of balance recovery ability: Maximum movement width of midpoint of the waist

A Mann-Whitney U test revealed that the KEL method showed a significantly larger maximum movement width than the MML method in the test of balance recovery ability (*p* = .012, Cliff's *d* = 0.65) (Table 3). This result supports hypothesis 3.

**Table 3:** The difference in maximum movement width in the test of balance recovery ability.

Test of balance recovery ability		Maximum movement width (m)	*p*	*Cliff's d*
KEL	*Median(IQR)*	0.73 (0.96-0.54)	0.012	0.65
MML		0.37 (0.16-0.65)		

KEL, Kinesthetic-Experiential Learning; MML, Model-Mastery Learning.

#### 3.3.4. Test of balance recovery ability: Success rates

A *χ*^2^ test revealed that the success rate of the KEL method was significantly higher than the MML method [*x*^2^ (1) = 33.27, *p* < .001, *Φ *= .24] (Table 4). This result supports hypothesis 3.

**Table 4:** The difference in success rates in the test of balance recovery ability.

Test of balance recovery ability	Success	Failure	Total	*χ*2	*Φ*
KEL	197	103	300	33.27***	0.24
	66%	34%	100%		
MML	117	163	280		
	42%	58%	100%		

KEL, Kinesthetic-Experiential Learning; MML, Model-Mastery Learning.

*** *p*<.001.

## 4. Discussion

This study compared the KEL and MML methods with regard to the difference between the movements while maintaining balance and the ability to recover balance. The results showed no difference between the learning methods in terms of the degree of difficulty of movement tasks or improvement in balancing time. Concerning RPE, the KEL method had significantly lower scores despite performing similar activities. It has been reported that RPE is affected not only by physiological exercise intensity but also by intrinsic motivation for exercise (22). A previous study confirmed that KEL increased intrinsic motivation for learning activities more than MML (13), and it is possible that this difference appeared as a difference in RPE scores.

There were differences in the actual movements made when maintaining balance and, in the ability, to recover balance. In the balance maintenance time test, interesting results were obtained concerning actual movements while maintaining balance. The most important result of this study was that the sway of head movements while maintaining balance differed between the two learning methods. Learners who practiced the balancing task using the KEL method showed larger head sways than those who used the MML method, while no significant difference was found between the two methods at the waist and knees. Furthermore, the stability of the waist is considered important for maintaining balance because the maximum sway width of the waist is about half that of the head and knees.

The total trajectory length of the head while maintaining balance was significantly longer with the KEL method, as was the sway of head movements, indicating that the head movement was more variable while maintaining balance with the KEL method than with the MML method. The maximum sway width indicates the maximum range of movement in the anteroposterior and mediolateral directions while maintaining balance. The total trajectory length indicates the movement within the maximum sway width. The present results indicated that in the KEL method, the learners maintained the balance while moving to stabilize the waist, whereas in the MML method, they maintained balance by keeping the entire body stationary to maintain the stability of the waist. These results support hypothesis 2. The compensatory postural adjustment is explained by multi-joint coordination underlying the contribution of various body segments to the recovery of stability (23). Moreover, good variability in balance control did not affect the position of the center of mass of the individual, while bad variability did (24). These results indicate that the learners' variability in the KEL method was sufficient.

Concerning the balancing time in the balance maintenance time test, time improved for both learning methods. These results support hypothesis 1. In other words, no difference was found between the learning methods, which may result from the number of practice sessions given to the participants. In contrast to the previous study by Matsuura et al. (13), which showed differences in the improvement in balancing time because of differences in learning methods, participants in the present study were given only one practice session instead of two. It should also be noted that, in the previous study, no difference between learning methods was seen when measurements were taken after the first session.; thus, no difference in learning methods in terms of the degree of improvement in balancing time was observed for shorter practice periods. However, with more practice, a difference between learning methods may be seen.

In the balance recovery test, the KEL method showed a larger maximum movement width at the midpoint of the waist and a higher success rate than the MML method. The one unique characteristic of this task was the inverse relationship between the movement width and the success rate: smaller movement width correlated with a higher rate of success, and greater movement width with a lower rate of success. As the movement width was more extensive and the success rate was higher for the KEL method than for the MML method, this result confirmed that the KEL method had wider limits of balance stability compared with the MML method and showed a higher ability to correct balance when participants were about to lose their balance. These results support hypothesis 3. Furthermore, in the balance recovery test, five trials in each direction were immediately performed without any practice time after the task was presented, which indicates that the learners' ability to adapt to new tasks is high while adopting the KEL method.

The one possible reason for the increased balance recovery ability in the KEL method as opposed to the MML method is that the KEL method included the practice of deliberately losing balance. Previous studies have confirmed that the experience of losing balance and perturbation training enhance the stability of one's balance (25, 26). The practice of intentionally falling from an exercise ball, which was one of the various experiences in the KEL method, may have enhanced the learners' dynamic stability on the ball, leading to an increased ability to recover their balance.

What is essential to the KEL method is the configuration of the learning environment. To give learners the experience of various motor sensations, including the experience of failure, a learning environment considering learners' safety from a physical and mental standpoint is essential to experience failure. Therefore, instructors are required to thoroughly ensure the safety of learners and create a learning environment in which learners can fail with peace of mind and without any physical risk. In this study, we created a learning environment by laying down soft mats and cushioning materials so that people could fail safely. As Gibson's (27) affordance theory states, various human actions, including physical exercise, are determined by the interaction between the body and the environment; therefore, it is important to create an environment that naturally draws out the movements of learners. This way of thinking, which regards experience as an interaction between the learner and the environment, has assumptions analogous to concrete experiences in the experiential learning model proposed by Kolb (28). In other words, as a future prospect, it may become possible to learn more effectively by incorporating the KEL method into the experiential learning model.

As a limitation of this study, only the early learning stages were examined. Thus, the results might be applicable to the early learning stages. In fact, learners who use significant degrees of volatility in their cognitive strategies at the beginning of task development can learn more effectively and are more likely to achieve tasks (29). Therefore, it is important to use the KEL method in the early stages of learning. Considering the whole stage of the learning process, there is a concept called the U-shaped curve in the progress of motor proficiency and variability (30). A U-shaped curve suggests high variability at the beginning of learning, which decreases as the learning progresses and increases again during the final stages of developing skilled performance. Based on this, future research should include the proper use of learning methods to measure long-term learning effects according to progress in exercise proficiency.

## 5. Conclusion

The present study aims to compare learners' movement variability while maintaining balance and the ability to recover balance using the kinesthetic-experiential learning (KEL) method of implicit learning and the model-mastery learning (MML) method of explicit learning. The results revealed that compensatory postural adjustments were carried out more often in KEL than in MML, although balancing time improved for both learning methods. Also, the KEL method showed a larger balance recovery width and a higher success rate than the MML method. These results suggest that using the KEL method as an implicit motor learning technique that makes use of invariant features at the initial stage of learning improves learners' balance recovery ability and increases movement variability while maintaining balance. As an outlook for the future, the flexible and adaptive strategy of the KEL method may effectively enhance performance among athletes who are required to adapt to different environments; thus, it would be worthwhile to change variables such as participants, learning tasks, and the learning period to examine their effects in the future.

## Data Availability

The raw data supporting the conclusions of this article will be made available by the authors, without undue reservation.
